# Rumen-Protected Niacin Supplementation Modulates Uterine Arterial Hemodynamics and Endometrial Microbiome Functional Potential in Postpartum Japanese Black Cattle

**DOI:** 10.3390/ani16203235

**Published:** 2026-10-14

**Authors:** Yuki Yorozui, Seiji Inoue, Yufei Mu, Shuichi Matsuyama, Satoshi Ohkura, Yasuhiro Morita

**Affiliations:** 1 Department of Animal Sciences, Graduate School of Bioagricultural Sciences, Nagoya University, Nagoya 464-8601, Japan; yorozui.yuki@c.mbox.nagoya-u.ac.jp (Y.Y.); inoue.seiji.s7@s.mail.nagoya-u.ac.jp (S.I.); shuichim@agr.nagoya-u.ac.jp (S.M.); saohkura@agr.nagoya-u.ac.jp (S.O.); 2 Department of Bioresource Sciences, Graduate School of Bioresource and Environmental Studies, Kyushu University, Fukuoka 819-0395, Japan; yufei.740@s.kyushu-u.ac.jp

**Keywords:** niacin, uterine environment, uterine hemodynamics, uterine microbiome

## Abstract

This study examined whether rumen-protected niacin (RPN) supplementation is associated with uterine hemodynamics and the postpartum uterine environment in Japanese Black cattle. Experiment 1 used a crossover design to evaluate acute uterine vascular responses to RPN in synchronized heifers (*n* = 6). Experiment 2 was an exploratory evaluation of postpartum cows (*n* = 6) during long-term RPN supplementation using uterine artery Doppler measurements, uterine inflammatory indicators, and endometrial microbiome analysis. In Experiment 1, high-dose RPN administration was associated with changes in uterine hemodynamic parameters (*p* < 0.05) and higher baseline-adjusted whole-blood NAD^+^ concentrations across the post-administration period (*p* < 0.001). In Experiment 2, RPN supplementation was associated with time-dependent differences in uterine hemodynamic parameters, whereas uterine inflammatory indicators were not significantly affected. Although broad treatment-associated differences in overall endometrial microbial diversity and community structure were not detected, exploratory differences in specific bacterial taxa were observed between treatment groups, and predicted metabolic pathways showed treatment-dependent temporal changes. Exploratory integrated analysis further identified concurrent within-animal patterns across uterine hemodynamics, microbial community structure, and predicted microbial functional potential. These exploratory findings suggest that RPN may modulate uterine hemodynamics and be associated with selective changes in the postpartum uterine environment, providing a basis for further investigation of the relationships among nutritional intervention, uterine hemodynamics, the endometrial microbial ecosystem, and subsequent reproductive physiology.

## Simple Summary

Rumen-protected niacin (RPN) is used as a nutritional supplement in cattle, but its potential effects on uterine circulation and the postpartum uterine environment remain unclear. This study investigated acute and longer-term responses to RPN supplementation in Japanese Black cattle. RPN administration was associated with changes in uterine hemodynamics and whole-blood NAD^+^ concentrations. During the postpartum period, RPN supplementation was also associated with changes in uterine hemodynamics and selective changes in the endometrial microbiome and its predicted functional potential. These findings suggest that nutritional modulation of uterine circulation may be associated with changes in the postpartum uterine environment and provide a basis for further investigation of the potential relationships between nutritional approaches and reproductive physiology in beef cattle.

## 1. Introduction

Adequate uterine perfusion is considered an important component of normal postpartum uterine involution, tissue repair, and the restoration of reproductive function [1]. Impaired uterine blood flow has been associated with delayed uterine recovery, persistent inflammation, and reduced fertility in cattle [2]. These observations suggest that uterine hemodynamics are closely associated with changes in the postpartum uterine environment. Previous studies have shown that uterine blood flow is a useful indicator of postpartum uterine recovery in cattle [3,4,5]. Transrectal Doppler ultrasonography has revealed characteristic changes in uterine arterial blood flow during the weeks following parturition, reflecting uterine involution and functional restoration [4]. Early postpartum studies in heifers have also demonstrated rapid adjustments in uterine artery diameter and blood flow velocity, indicating dynamic regulation of uterine perfusion during this period [5]. In addition, oxytocin-induced uterine contractions are accompanied by marked changes in uterine blood flow, further highlighting the close link between uterine perfusion and uterine function [3]. Together, these findings support the use of uterine hemodynamics as a physiological marker of postpartum uterine recovery.

During the postpartum period, the uterus undergoes dynamic microbial colonization followed by progressive clearance as tissue repair proceeds [6]. Alterations in the composition of the endometrial microbiome have been linked to uterine inflammation and impaired reproductive performance [7]. Longitudinal studies in dairy cows have shown that the endometrial microbiota changes markedly from early to later postpartum stages and is closely associated with endometrial gene expression profiles related to tissue repair, immune regulation, and the resumption of ovarian cyclicity [8]. Host physiological conditions, including tissue perfusion and oxygen availability, are likely to influence microbial growth and community structure within the uterine lumen. Consistent with this, disruption of normal postpartum microbial succession—such as delayed differentiation between the vaginal and uterine microbiomes—has been associated with endometritis and delayed uterine recovery [9], while metritis-associated microbiomes have been linked to long-term alterations in endometrial gene expression and reduced fertility [10]. However, the extent to which changes in uterine hemodynamics contribute to modulation of the endometrial microbiome during the postpartum period has not been well characterized, particularly in the context of nutritional interventions that may influence uterine physiology. Moreover, whether niacin supplementation is associated with changes in the postpartum endometrial microbiome has not been established.

Agricultural production is expected to be increasingly affected by rising ambient temperatures associated with global warming. In dairy and beef cattle production, maintaining reproductive performance and milk production under heat stress has therefore become a major concern. Niacin, a water-soluble vitamin, has been used in rumen-protected form as a nutritional approach to alleviate heat stress, and several studies have reported increased milk yield and reduced body temperature in dairy cows during summer. In cattle, supplementation with rumen-protected niacin (RPN) has been shown to improve milk production and dry matter intake under hot environmental conditions [11,12,13]. In contrast, negative effects of niacin supplementation on milk yield and milk composition have also been reported [14]. Taken together, these findings indicate that the physiological actions of niacin in cattle are not fully understood, particularly with respect to its effects beyond thermoregulation.

Niacin induces vasodilation through activation of the GPR109A receptor, which is expressed on skin Langerhans cells and mediates prostaglandin-dependent dilation of peripheral capillaries, facilitating heat dissipation. Expression of GPR109A has also been detected in the uterus and ovaries of cattle [15], suggesting that niacin may influence blood flow in reproductive organs. However, the functional role of this receptor in uterine tissues of ruminants has not been clarified. In humans and experimental models, excessive niacin intake is known to cause marked peripheral vasodilation accompanied by skin flushing and discomfort [16], mediated by the arachidonic acid cascade. Whether similar vascular mechanisms operate in the bovine uterus, and whether such responses are beneficial or detrimental during the postpartum period, remains to be determined.

It is unknown whether RPN supplementation can modulate uterine hemodynamics in the postpartum period. It is also unclear whether such hemodynamic changes are associated with alterations in the endometrial environment, including microbial composition and predicted functional potential. Therefore, we hypothesized that RPN supplementation modulates uterine hemodynamics and is associated with changes in the postpartum uterine environment in beef cattle. To test this hypothesis, we investigated the effects of RPN supplementation on uterine hemodynamics and evaluated postpartum uterine status by endometrial cytology and microbiome profiling in Japanese Black cows. The aim of this study was to determine whether RPN-associated changes in uterine hemodynamics are accompanied by changes in the postpartum uterine environment, including alterations in the endometrial microbiome and its predicted functional potential, and to explore the potential relationships among nutritional intervention, uterine hemodynamics, the postpartum endometrial environment, and subsequent reproductive physiology in beef cattle.

## 2. Materials and Methods

### 2.1. Animals

The experiments were conducted at the university farm of the Field Science Center, Graduate School of Bioagricultural Sciences, Nagoya University (35°6′42″ N, 137°4′57″ E), located in Togo, Japan. Six synchronized, healthy Japanese Black heifers (10–14 month of age, BCS 3.5) and six clinically healthy postpartum cows (3–7 years of age, parity 2–4, BCS 3.25–3.5) were assigned to Experiments 1 and 2, respectively. All animals were housed under standard conditions and provided with water ad libitum from individual tanks. All animals were acclimated to the housing and feeding conditions for at least one week before the start of each experiment. Animals were monitored daily, and no animals were excluded because of health deterioration. The postpartum cows had no clinically apparent reproductive disorders at enrollment.

### 2.2. Animal Ethics

All experimental procedures were approved by the Committee of Care and Use of Experimental Animals of the Graduate School of Bioagricultural Sciences, Nagoya University (approval number: AGR2020061). All animal experiments were performed in accordance with the relevant guidelines and regulations. The study is reported in accordance with the ARRIVE guidelines (https://arriveguidelines.org, accessed on 2 September 2026). No animals were euthanized during or after the experiment. All cows were returned to the experimental farm and maintained under standard husbandry conditions after the completion of the study. Sample size was determined based on ethical considerations and feasibility constraints, consistent with exploratory physiological studies in large animals; no formal a priori power calculation was performed.

### 2.3. Experimental Design

Experiment 1 served as an acute proof-of-concept experiment to determine whether RPN could elicit measurable changes in uterine hemodynamics, whereas Experiment 2 explored the effects of chronic RPN supplementation under postpartum conditions.

#### 2.3.1. Experiment 1: Acute Effects of RPN Administration on Uterine Artery Hemodynamics

**Figure 1:**
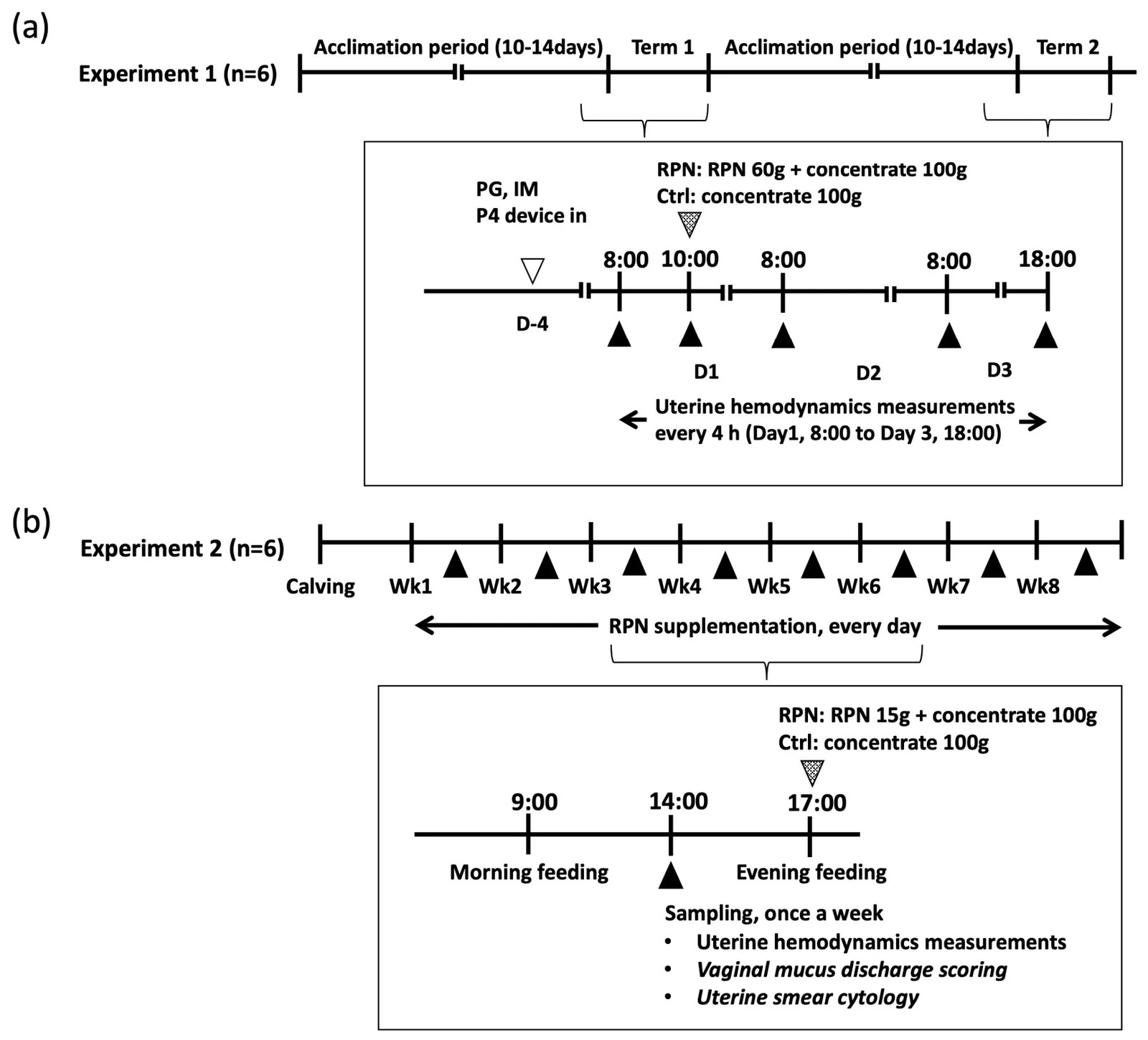
Experimental design of the RPN administration studies. (**a**) Experiment 1: Acute effects of rumen-protected niacin (RPN) on uterine arterial hemodynamics. Six estrous-synchronized Japanese Black heifers were studied in a randomized crossover design. Each animal received a single oral dose of RPN (60 g) or control concentrate (Ctrl), with a washout period between treatments. Uterine arterial hemodynamics was measured by transrectal Doppler ultrasonography every 4 h from 2 h before to 46 h after administration. (**b**) Experiment 2: Effects of postpartum RPN supplementation on uterine physiology. Six multiparous cows (1 week postpartum) were assigned to RPN or control (Ctrl) groups (*n* = 3 per group). RPN (15 g/day) was provided from weeks 1–8 postpartum. Uterine arterial hemodynamics was assessed weekly by Doppler ultrasonography, and vaginal discharge scoring and endometrial cytology were used to evaluate uterine involution, inflammatory status, and uterine microbiome profiles.

Experiment 1 was conducted between March and July 2020 using six synchronized Japanese Black heifers. Estrous cycles were synchronized with an intravaginal progesterone device (Ovapron V, Kyoritsu Seiyaku Corporation, Tokyo, Japan) during the luteal phase. The same synchronization protocol was applied before each experimental period to minimize variation in reproductive status between the two periods. Animals were housed under controlled wind and sunlight conditions and tied in groups of three at 1-m intervals. Each animal received both treatment in a randomized crossover design: 60 g of RPN (Niacoat, Nissan Gosei, Tokyo, Japan; containing 31 g niacin) mixed with 100 g of concentrate (RPN) or 100 g of concentrate alone (Ctrl), with a two-week washout period between treatments (Figure 1a). Three heifers received RPN followed by Ctrl, whereas the remaining three received Ctrl followed by RPN, thereby balancing treatment order across animals. The around two-week washout period was included to minimize potential carryover effects between experimental periods. Treatments were individually offered, and complete consumption was visually confirmed. Animals received the same basal diet from the same feed lot throughout the experiment. To minimize variation in dietary vitamin intake, all heifers received the same rice-straw-based basal diet throughout the experimental period (4 kg of rice straw and 400 g of concentrate per animal per day). Uterine artery blood-flow parameters were recorded at 4 h intervals from 2 h before to 46 h after each administration.

#### 2.3.2. Experiment 2: Effect of Postpartum RPN Feeding on Uterine Physiology

Experiment 2 was conducted between September 2020 and January 2021 using six multiparous cows enrolled at one week postpartum. Each cow was separated from its calf and housed individually (Figure 1b). All cows were fed a mixed diet of ryegrass straw and timothy grass silage (6 kg per animal per day in total) twice daily at approximately 09:00 h and 16:00 h, with ad libitum access to water. Cows were randomly assigned to the RPN or control group (*n* = 3 per group). Baseline measurements and sample collection at week 1 (8–12 days after calving) were performed before the first RPN administration. The RPN supplementation group received 15 g of RPN (equivalent to 7.75 g niacin) mixed with 100 g of concentrate once daily from 1 to 8 weeks postpartum (RPN), while the control group received only 100 g of concentrate alone (Ctrl). The 15 g daily dose was selected within the manufacturer-recommended supplementation range of 12–20 g/day. RPN was individually offered with a small amount of concentrate to ensure complete consumption. Animals within the experiment received the same basal diet from the same feed lot. Uterine arteries were examined weekly at 1400 h, corresponding to 5–6 h after morning feeding, by transrectal Doppler ultrasonography using the same procedures as in Experiment 1. Vaginal discharge was assessed weekly, and endometrial cytology was performed at weeks 1, 4, and 8 postpartum to monitor the progression of uterine involution and postpartum inflammation.

#### 2.3.3. Blood Sampling for Measuring Whole-Blood NAD^+^

In Experiment 1, blood samples were collected from the jugular vein at 2 h intervals from 2 h before to 46 h after RPN administration. NAD^+^ concentrations were measured in whole blood by a commercial laboratory (Nissan Gosei, Tokyo, Japan) using an enzymatic colorimetric assay. Briefly, NAD^+^ was extracted from whole blood using NAD/NADP extraction buffer followed by heat treatment at 90 °C for 90 s and centrifugation. NAD^+^ concentrations were subsequently determined using an alcohol dehydrogenase (ADH)-coupled reaction with MTT and phenazine methosulfate (PMS), and absorbance was measured at 570 nm. Concentrations were calculated against an NAD^+^ standard curve. NAD^+^ concentrations were measured only in Experiment 1 to evaluate the acute systemic response to RPN administration.

#### 2.3.4. Observation of Uterine Arterial Hemodynamics

In both experiments, the external iliac artery, external iliac vein, and uterine artery were identified transrectally according to the method of Ando et al. (2007) [17]. In Experiment 1 and 2, uterine artery diameter (UAD), resistance index (RI), pulsatility index (PI), and time-averaged maximum flow velocity (TAMV) were measured by transrectal Doppler ultrasonography using a portable ultrasound system (S6V, SonoScape Medical Corp., Shenzhen, China) equipped with a 5 MHz linear probe. For pulsed-wave Doppler measurements, the Doppler frequency was set at 4.5 MHz, with a pulse repetition frequency of 6.0 kHz and a wall filter of 150 Hz. Imaging depth, Doppler gain, and sample volume were adjusted as necessary to obtain a clear and stable uterine arterial waveform. The transducer was positioned to visualize the uterine artery in a standardized orientation, with the vessel course maintained consistently relative to the ultrasound image. This standardized imaging plane was used throughout the measurements to minimize variation in the Doppler insonation angle. Doppler angle correction was not applied. Measurements were averaged over five consecutive cardiac cycles. Doppler measurements were performed by the same experienced operator using consistent anatomical landmarks and acquisition procedures throughout the study, and recorded images were analyzed offline by another investigator using standardized measurement criteria.

#### 2.3.5. Examination in Vaginal Mucus Discharge and Uterine Smear Cytology

In Experiment 2, vaginal mucus was collected weekly at 1400 h (5–6 h after morning feeding) using a Metricheck device (Simcro Tech Ltd., Hamilton, New Zealand). Samples were scored on a 5 point scale (0–4) based on the Purulent vaginal discharge (PVD) scores, following Sheldon et al. (2006) [18]. Vaginal discharge scoring was performed by the same trained examiner throughout the study.

Endometrial cytology was performed at weeks 1, 4, and 8 postpartum using a cytobrush (Metribrush, Fujihira Industry Co., Tokyo, Japan), as described previously [19]. To minimize vaginal and cervical contamination, the Metribrush was introduced into the uterus within a protective artificial-insemination sheath, and the brush was exposed from its outer cylinder only after reaching the uterine lumen and retracted after sampling. Cytobrushes were rolled onto glass microscope slides and immediately fixed with Cytokeep II (Alfresa Pharma Co., Osaka, Japan). Slides were stained with Diff-Quik (Sysmex, Kobe, Japan) for 20 s. A minimum of 200 cells per slide was evaluated at 400× magnification, and the percentage of polymorphonuclear leukocytes (PMN%) was calculated as an index of endometrial inflammation. All slides were independently assessed by two clinicians, one of whom was blinded to the sampling and treatment information, following Wagener et al. (2017) [20].

After cytological sampling, the cytobrush tip was rinsed in sterile physiological saline to recover residual cellular material and uterine secretions. The saline suspension was centrifuged at high speed (10,000× *g* for 10 min) to pellet endometrial cells, inflammatory cells, and adherent bacteria. Pellets were stored at −30 °C until further microbiome analysis.

#### 2.3.6. DNA Extraction for Endometrial Microbiome Analysis

For endometrial microbiome assessment, the cell pellets collected at weeks 1 and 4 were used. Week 4 was selected as the follow-up sampling point to characterize changes in the endometrial microbiome during the period associated with the resumption of postpartum ovarian cyclicity, rather than at the later week 8 time point. DNA extraction, library preparation, 16S rRNA gene sequencing, and bacterial taxonomic profiling were performed by Seibutsu Giken Co. (Kanagawa, Japan). Detailed procedures for sample processing, DNA extraction, V3–V4 amplicon library preparation, sequencing, and bioinformatic processing are provided in the Supplementary Methods.

Raw sequencing data have been deposited in the NCBI Sequence Read Archive (SRA) under BioProject accession number PRJNA1431663.

### 2.4. Statistical Analysis

Statistical analyses were performed using R version 4.3.2 (R Foundation for Statistical Computing, Vienna, Austria). All tests were two-sided, with *p* < 0.05 considered statistically significant unless otherwise specified. Model assumptions were assessed using appropriate diagnostic procedures. Cow identity was included as a random effect in mixed-effects models to account for repeated measurements within animals. Details of individual analyses are provided below.

#### 2.4.1. Whole-Blood NAD^+^ Concentration

Changes in whole-blood NAD^+^ concentrations were calculated relative to the pre-administration value at −2 h (ΔNAD^+^). Post-administration ΔNAD^+^ values (0–46 h) were analyzed using a linear mixed-effects model with treatment, time, treatment × time interaction, and experimental period as fixed effects and cow identity as a random effect. Time was treated as a categorical variable. Estimated marginal means were used to evaluate the overall treatment effect.

#### 2.4.2. Uterine Arterial Hemodynamics Analysis

In Experiment 1, changes in uterine arterial Doppler parameters (UAD, TAMV, RI, PI, and HR) were calculated relative to the pre-administration value at −2 h, and baseline-adjusted values (Δ) from −2 to 46 h were analyzed with treatment, time, treatment × time interaction, and experimental period as fixed effects. In Experiment 2, changes in UAD, TAMV, RI, and PI were calculated relative to the pre-supplementation value at week 1, and baseline-adjusted values (Δ) from weeks 1 to 8 were analyzed with treatment, week, and treatment × week interaction as fixed effects. For both experiments, linear mixed-effects models with cow identity as a random effect were fitted using maximum likelihood estimation. Time and week were treated as categorical variables, and fixed effects were evaluated by Type III ANOVA with Satterthwaite’s approximation. For Experiment 1, estimated marginal means with 95% confidence intervals were calculated for the overall treatment differences.

#### 2.4.3. Purulent Vaginal Discharge (PVD)

PVD scores were recorded longitudinally after calving to assess clinical signs associated with postpartum reproductive tract inflammatory status. Given the small number of animals in each treatment group and the ordinal nature of the PVD scores (0–4), PVD scores were evaluated descriptively based on their longitudinal trajectories rather than by formal statistical inference. Individual PVD scores were plotted over the postpartum period to visualize temporal changes within each animal and treatment group.

#### 2.4.4. Polymorphonuclear Leukocytes (PMN%)

PMN% is adequate as diagnostic criterion for endometritis and PMN% < 4, 5 < PMN% < 14, 15 < PMN% are categorized as normal, sub-clinical endometritis, and clinical endometritis, respectively [19]. PMN% data were treated as a continuous proportional outcome and analyzed using generalized linear mixed-effects models implemented in the *glmmTMB* package in R. Because PMN% represents a bounded proportion (0–100%), values were converted to proportions and modeled assuming a beta distribution with a logit link function. Treatment, postpartum week (weeks 1, 4, and 8; categorical), and their interaction were included as fixed effects, and cow identity was included as a random intercept to account for repeated measurements within individuals. The significance of fixed effects was evaluated using Type III Wald chi-square tests.

#### 2.4.5. Microbiome Analysis

One control sample at week 1 was lost prior to microbiome analysis and was therefore not included. Consequently, microbiome analyses at week 1 included three RPN cows and two Ctrl cows at week 1, and three cows per group at week 4. Longitudinal analyses were therefore performed using animals with paired samples at weeks 1 and 4 (RPN, *n* = 3; Ctrl, *n* = 2), with week 1 serving as the pre-supplementation baseline. Alpha diversity was assessed using the Shannon index, Chao1 richness, and Pielou’s evenness. For each index, within-animal changes from week 1 to week 4 (Δ = week 4 − week 1) were compared between treatments using two-sided exact permutation tests. Beta diversity was assessed using principal coordinates analysis (PCoA; via cmdscale) based on robust Aitchison distances (vegdist, method = “robust.aitchison”) using all available samples [21,22,23]. For longitudinal comparison, robust centered log-ratio (rCLR)-transformed abundances were used to calculate within-animal changes from week 1 to week 4 (ΔrCLR). Euclidean distances among ΔrCLR vectors were calculated, and treatment-associated differences in community-level changes were evaluated by PERMANOVA using exact permutation testing.

Exploratory differential abundance analysis of microbial taxa was conducted using DESeq2 (version 1.42.0) in R [24]. For this analysis, the ASV table was taxonomically aggregated at the genus level, and the resulting genus-level abundance table was used as input for DESeq2. Samples were classified into four treatment-by-time groups (RPN-week 1, Ctrl-week 1, RPN-week 4, and Ctrl-week 4), which were included as a single design factor. Wald tests were used to evaluate pairwise contrasts between RPN and Ctrl at each postpartum week. These comparisons were cross-sectional. *p*-values were adjusted using the Benjamini–Hochberg false discovery rate, and taxa with adjusted *p* < 0.05 and |log2 fold change| > 1 were considered differentially abundant.

#### 2.4.6. PICRUSt2 Analysis

Predicted functional metabolic pathways of the endometrial microbiome were generated using PICRUSt2 (q2-picrust2, QIIME2 v2024.5) [25,26]. The ASV-level feature table and corresponding representative sequences generated by the QIIME 2 pipeline were directly used as input for PICRUSt2. ASVs with insufficient alignment to reference sequences (min_align = 0.8) were excluded (*n* = 53), and only one ASV exceeded the default nearest sequenced taxon index (NSTI) cutoff (2.0) and was removed. Predicted pathway abundances were analyzed using linear mixed-effects models with treatment, postpartum week, and their interaction as fixed effects and cow as a random intercept with the lme4 package in R. Models were fitted by maximum likelihood. *p*-values for treatment × week interactions were adjusted across pathways using the Benjamini–Hochberg false discovery rate (FDR) procedure. Given the exploratory nature of this analysis, pathways with FDR-adjusted *p* < 0.05 were considered to show evidence of treatment-dependent temporal differences in predicted functional potential.

#### 2.4.7. Exploratory Integrative Analysis of Uterine Blood Flow, Microbiome Structure, and Predicted Metabolic Pathways

An exploratory, hypothesis-generating integrative analysis was performed within the RPN group to examine associations among uterine blood flow, microbiome structure, and predicted metabolic functions. RI was used as the representative blood-flow parameter, and microbiome structure was represented by PCoA Axis 1 based on robust Aitchison distance. One predicted pathway (PWY-5181), which showed an FDR-adjusted treatment × week interaction and a consistent direction of change among individual animals within each treatment group, was selected as the representative predicted functional pathway. Changes from week 1 to week 4 (Δ = week 4 − week 1) were calculated to reduce inter-individual variability, and the relationships among ΔRI, ΔPCoA Axis 1, and Δpathway were examined descriptively within the RPN group. Given the very small sample size (*n* = 3), no formal statistical inference was performed, and the analysis was considered exploratory and hypothesis-generating.

## 3. Results

### 3.1. Experiment 1

#### 3.1.1. Whole-Blood NAD^+^ Concentrations

**Figure 2:**
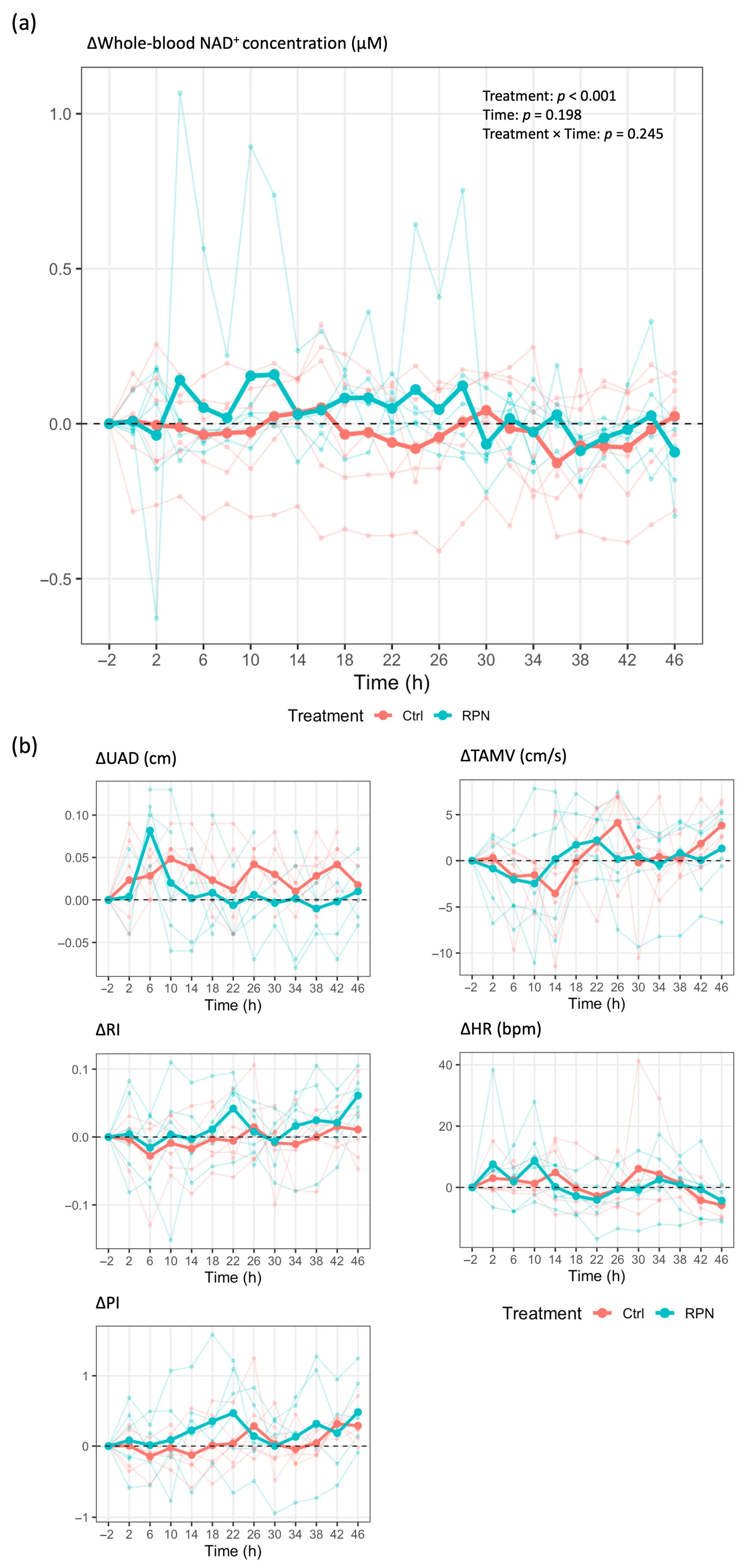
Acute effects of RPN on whole blood NAD^+^ concentration and uterine arterial hemodynamics. (**a**) Baseline-adjusted changes (Δ) in whole-blood NAD^+^ concentration measured at 2 h intervals from 2 h before to 46 h after administration. (**b**) Baseline-adjusted changes (Δ) in uterine artery diameter (UAD), resistance index (RI), pulsatility index (PI), time-averaged maximum velocity (TAMV), and heart rate (HR) were measured by transrectal Doppler ultrasonography at 4 h intervals from 2 h before to 46 h after a single oral administration (*n* = 6). Each animal received both treatments (Ctrl and RPN) in separate experimental sessions with washout period. Red and blue lines represent the control (Ctrl) and RPN treatments, respectively.

Whole-blood NAD^+^ concentrations showed substantial inter-individual variability. After adjustment to the pre-administration value at −2 h, ΔNAD^+^ was higher during RPN treatment than during the control treatment over the post-administration period (treatment effect, *p* < 0.001; Figure 2a). Neither time (*p* = 0.198) nor the treatment × time interaction (*p* = 0.245) was significant. Whole-blood NAD^+^ was assessed only in Experiment 1 and was not measured in the postpartum cows in Experiment 2.

#### 3.1.2. Effects of High-Dose Niacin Supplementation on Uterine Arterial Hemodynamics

No anorexia or other behavioral changes were observed in any heifer throughout the experimental period. Baseline-adjusted uterine arterial Doppler parameters (Δ; each time point relative to −2 h) were evaluated using linear mixed-effects models (Figure 2b). Uterine artery diameter (UAD) showed significant effects of treatment (*p* = 0.015) and time (*p* = 0.009), whereas the treatment × time interaction did not reach statistical significance (*p* = 0.080). Resistance index (RI) showed significant effects of treatment (*p* = 0.006) and time (*p* = 0.026), whereas the treatment × time interaction was not significant (*p* = 0.759). Pulsatility index (PI) exhibited a significant effect of treatment (*p* = 0.012), whereas the effects of time (*p* = 0.060) and the treatment × time interaction (*p* = 0.600) were not significant. Time-averaged maximum velocity (TAMV) showed a significant effect of time (*p* = 0.017), but neither the treatment effect (*p* = 0.556) nor the treatment × time interaction (*p* = 0.558) was significant. Heart rate (HR) also showed a significant effect of time (*p* = 0.018), whereas neither the treatment effect (*p* = 0.938) nor the treatment × time interaction (*p* = 0.708) was significant.

### 3.2. Experiment 2

No diseases known to affect uterine recoveries, such as dystocia, postpartum retardation, or postpartum fever, were observed in any of six cows tested in this experiment. All newborn calves were vigorous and healthy during the first week postpartum until cow–calf separation.

#### 3.2.1. Chronic Effects of RPN Supplementation on Uterine Arterial Hemodynamics

**Figure 3:**
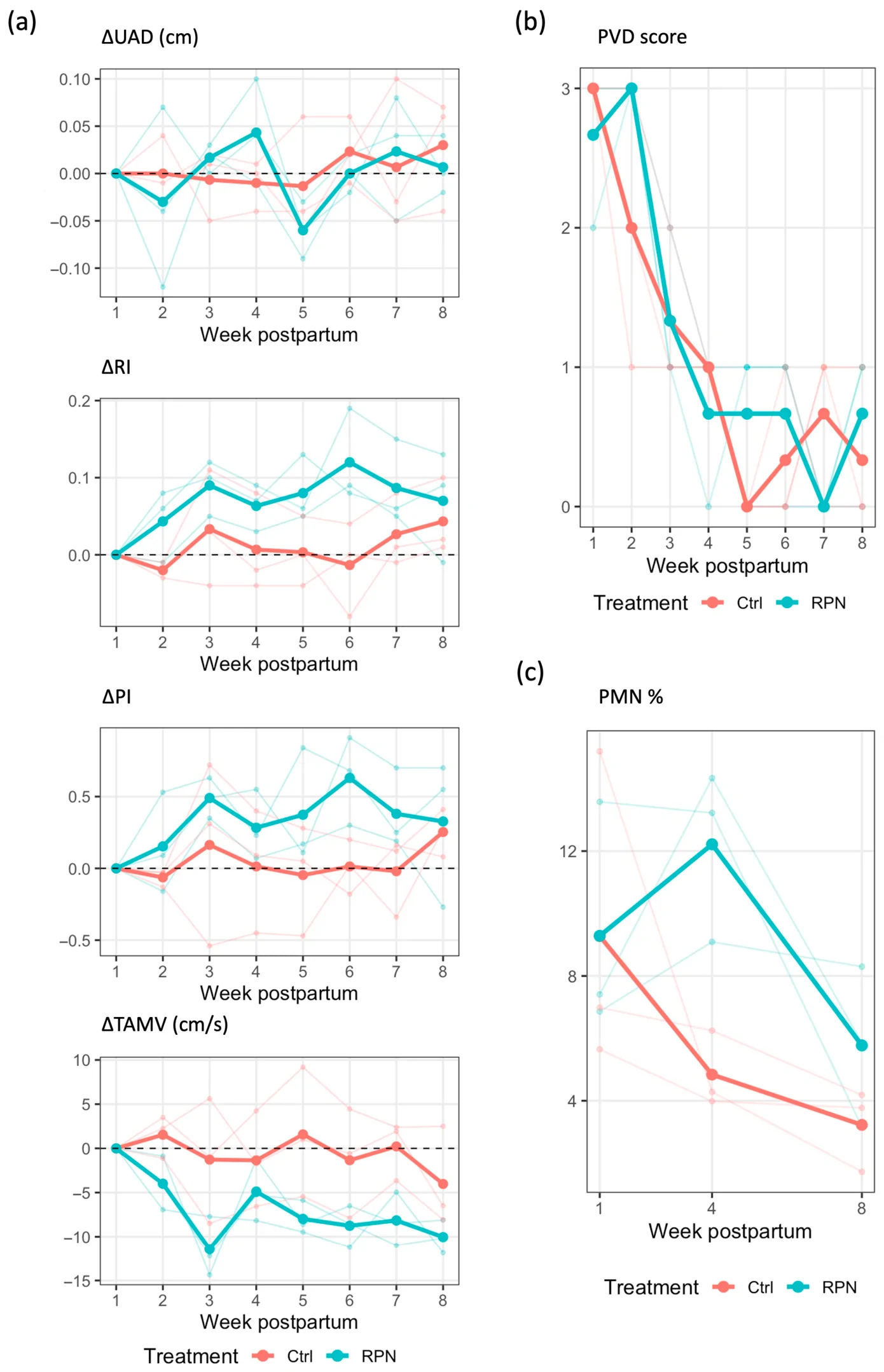
Chronic effects of RPN supplementation on postpartum uterine arterial hemodynamics and inflammatory indicators. (**a**) Baseline-adjusted weekly changes (Δ relative to week 1) in uterine artery diameter (UAD), time-averaged maximum velocity (TAMV), resistance index (RI), and pulsatility index (PI) measured by transrectal Doppler ultrasonography (*n* = 3 per group). (**b**) Purulent vaginal discharge (PVD) scores. (**c**) Endometrial polymorphonuclear leukocyte (PMN) proportions. Red and blue lines represent the control (Ctrl) and RPN groups, respectively.

Baseline-adjusted changes (Δ; each week relative to week 1) in uterine arterial hemodynamics parameters were analyzed using linear mixed-effects models (Figure 3a). UAD showed no significant effects of treatment, week, or their interaction (Treatment: *p* = 0.84; Week: *p* = 0.13; Treatment × Week: *p* = 0.27). In contrast, TAMV was significantly affected by treatment (*p* = 0.020), week (*p* < 0.001), and their interaction (*p* = 0.018). RI showed a treatment effect of *p* = 0.057 and significant effects of week (*p* = 0.0015) and the treatment × week interaction (*p* = 0.013). For PI, neither treatment (*p* = 0.101), week (*p* = 0.061), nor the treatment × week interaction (*p* = 0.237) was significant.

#### 3.2.2. Vaginal Mucus Score and Uterine Smear Cytology

Changes in Purulent vaginal discharge (PVD) and endometrial polymorphonuclear leukocyte (PMN) proportions are shown in Figure 3b and Figure 3c, respectively. At week 1, all three control cows had a PVD score of 3, whereas the RPN cows had scores of 2–3. By week 3, scores had decreased to 1–2 in all cows, and by week 5, all cows in both groups had scores of 0 or 1. Thereafter, PVD scores remained predominantly at 0 or 1 through week 8 in both groups. Endometrial PMN percentage, analyzed as a continuous inflammatory indicator using a beta-distributed generalized linear mixed-effects model with a logit link. For endometrial PMN proportions, a significant effect of postpartum week was detected (*p* = 0.005), whereas neither the treatment effect (*p* = 0.861) nor the treatment × week interaction (*p* = 0.051) was statistically significant.

#### 3.2.3. Endometrial Microbiome

**Figure 4:**
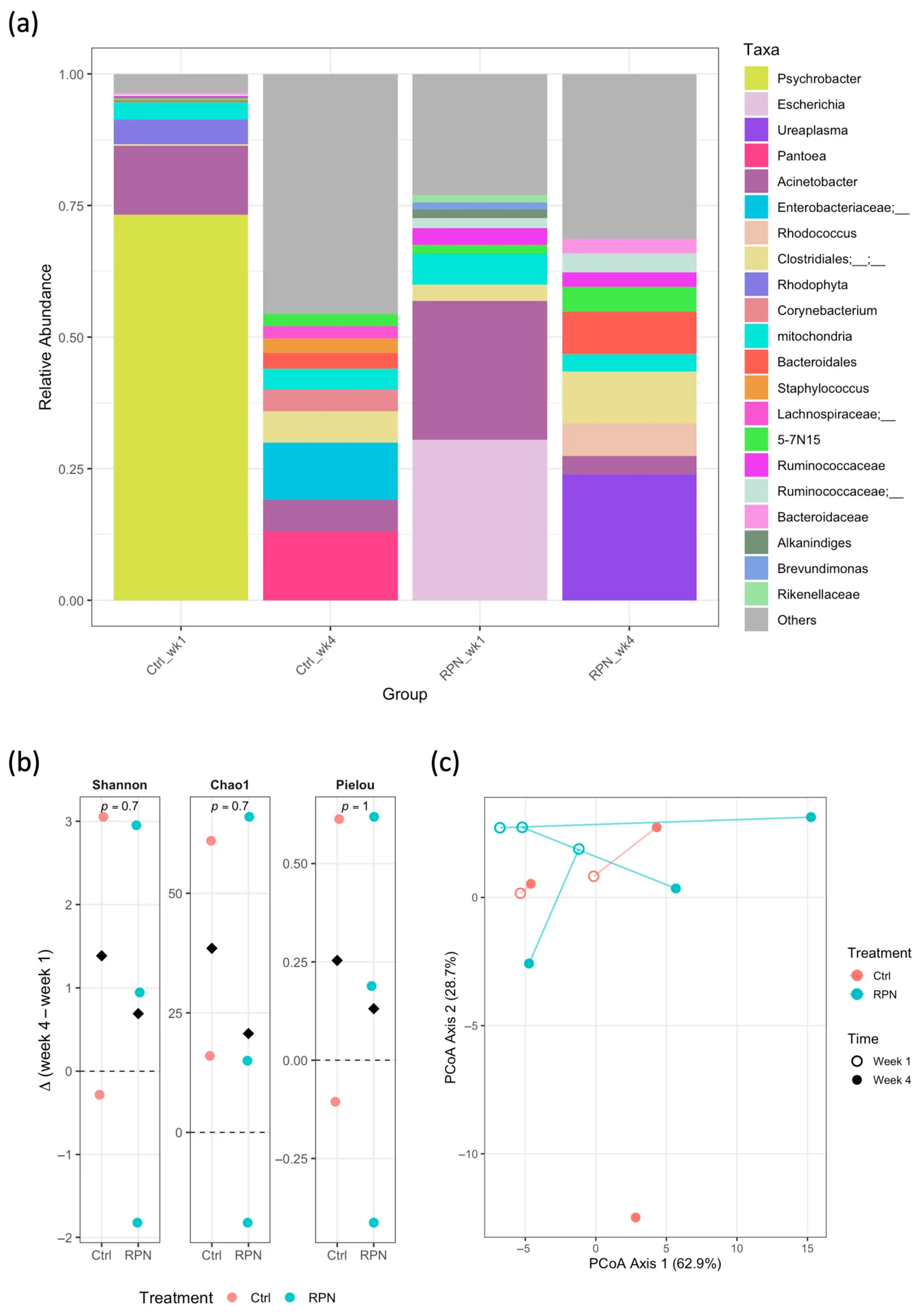
Endometrial microbiome composition and diversity during postpartum RPN supplementation. (**a**) Relative abundance of the 10 most prevalent bacterial taxa across treatment groups (Ctrl and RPN) and postpartum weeks (week 1: RPN, *n* = 3; Ctrl, *n* = 2; week 4: RPN, *n* = 3; Ctrl, *n* = 3). (**b**) Alpha-diversity indices (Shannon, Chao1, and Pielou). Treatment effects on changes from week 1 to week 4 were evaluated using an exact permutation test. (**c**) Beta-diversity based on robust Aitchison distances visualized by principal coordinate analysis (PCoA). Treatment effects on changes in microbial community structure from week 1 to week 4 were evaluated using exact PERMANOVA.

Figure 4a summarizes the relative abundances of the 10 most prevalent bacterial taxa across treatment groups (Ctrl and RPN) and postpartum time points (week 1 and week 4). Across all samples, the dominant taxa were *Acinetobacter* (11.8%), *Escherichia* (8.55%), *Psychrobacter* (7.45%), *Ureaplasma* (7.18%), *Clostridiales* (6.32%), *Bacteroidales* (4.01%), *Pantoea* (3.57%), *5–7N15* (3.13%), *Enterobacteriaceae* (3.08%), and *Ruminococcaceae* (2.82%).

Alpha-diversity metrics are shown in Figure 4b. Baseline-adjusted changes in alpha diversity from week 1 to week 4 did not differ significantly between the RPN and control groups for the Shannon index (exact *p* = 0.700), Chao1 richness (exact *p* = 0.700), or Pielou’s evenness (exact *p* = 1.000). Beta-diversity analysis based on robust Aitchison distances is presented in Figure 4c. Similarly, the baseline-adjusted change in overall microbial community composition, assessed using ΔrCLR vectors, did not show a detectable treatment-associated difference (PERMANOVA, R^2^ = 0.085, pseudo-F = 0.278, exact *p* = 1.000).

**Figure 5:**
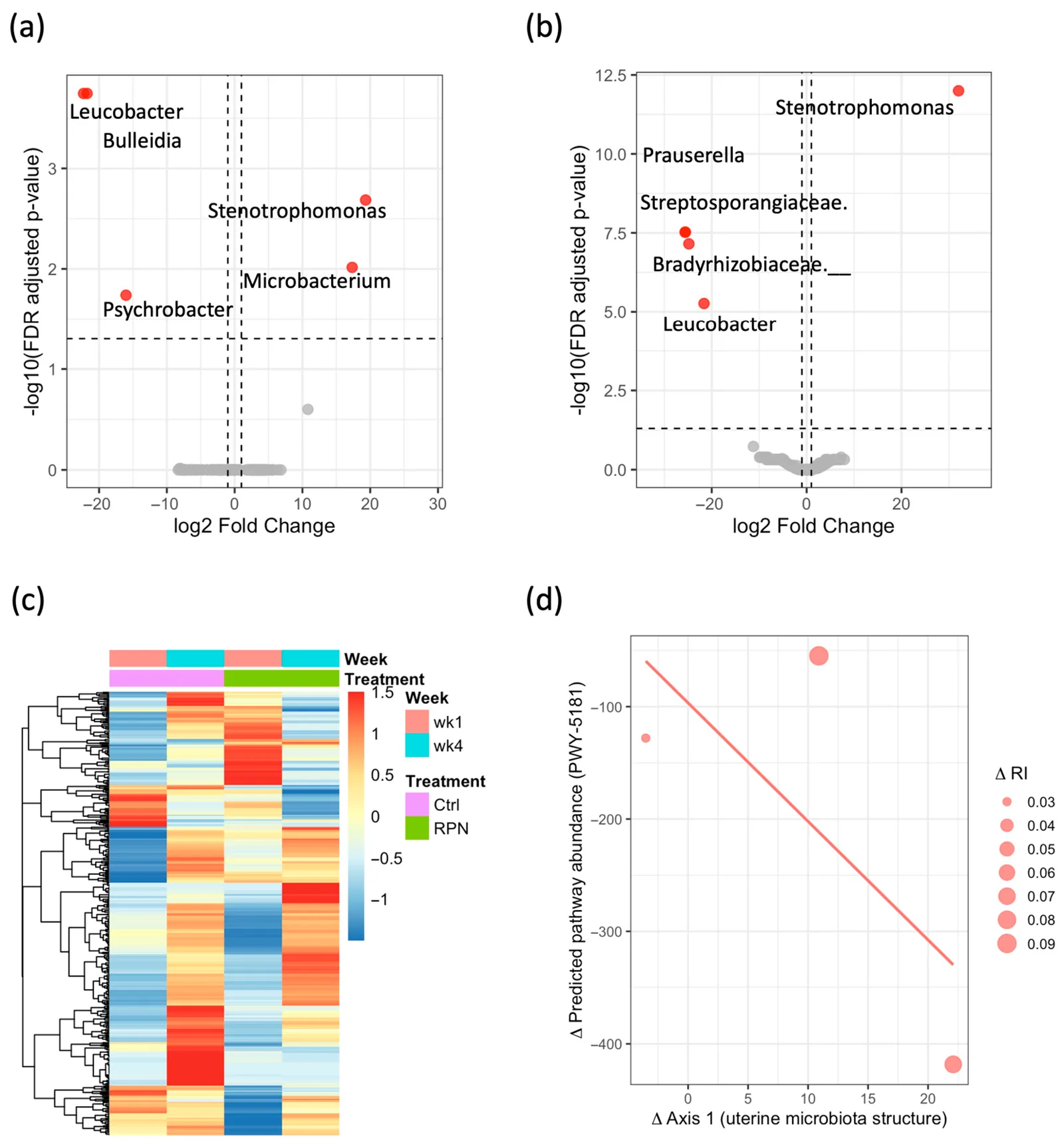
Differential taxonomic, functional, and integrated analyses of the endometrial microbiome. (**a**,**b**) Differential abundance of bacterial taxa between Ctrl and RPN groups at week 1 (pre-supplementation baseline, (**a**) and week 4 (**b**), identified using DESeq2. Selected taxa showing exploratory candidate differential-abundance signals in RPN are displayed (FDR-adjusted *p* < 0.05, |log_2_ fold change| > 1). (**c**) Predicted functional pathway profiles inferred by PICRUSt2 and analyzed using linear mixed-effects models. Pathways showing treatment × week interaction effects (FDR-adjusted *p* < 0.05) are indicated. (**d**) Integrated analysis in RPN-treated cows showing associations among changes (Δ; week 4 − week 1) in uterine arterial resistance index (RI), microbial community structure (PCoA Axis 1), and predicted metabolic pathway abundances.

Exploratory differential abundance analysis between Ctrl and RPN samples at weeks 1 and 4 was performed using DESeq2. Exploratory candidate differential-abundance signals were identified at both sampling points despite the absence of detectable treatment-associated differences in overall community composition. Because week 1 was the pre-supplementation baseline, the differences observed at week 1 represent pre-existing between-group differences rather than effects of RPN supplementation. At week 1 (Figure 5a), *Stenotrophomonas* and *Microbacterium* showed higher estimated abundances in RPN than in Ctrl (log_2_ fold change = 19.32 and 17.34; adjusted *p* = 0.0021 and 0.0097, respectively). In contrast, *Psychrobacter*, *Bulleidia*, and *Leucobacter* showed lower estimated abundances in RPN (log_2_ fold change = −16.02, −21.76, and −22.29; adjusted *p* = 0.018 and <0.01, respectively). At week 4 (Figure 5b), *Stenotrophomonas* showed a large positive estimated log_2_ fold change in RPN (log_2_ fold change = 32.03, adjusted *p* = 1.0 × 10^−12^), whereas members of *Streptosporangiaceae*, *Prauserella*, *Bradyrhizobiaceae*, and *Leucobacter* showed negative estimated log_2_ fold changes (adjusted *p* < 1 × 10^−7^). Inspection of individual-sample counts indicated that several of these candidate taxa were sparsely detected, with some occurring in only one or a few animals (Table S1). Therefore, the large estimated log_2_ fold changes should be interpreted cautiously and are reported here as exploratory candidate differential-abundance signals rather than as estimates of biological effect magnitude.

#### 3.2.4. Predicted Functional Metabolic Pathways of Endometrial Microbiota

Exploratory PICRUSt2 analysis predicted functional pathway profiles across the four groups (Figure 5c), identifying 403 pathways in total. To evaluate treatment- and time-dependent effects, linear mixed-effects models were applied to each pathway with treatment, week, and their interaction as fixed effects and cow identity as a random effect. After false discovery rate correction, two pathways (PWY-5181 and PWY-7431) showed significant treatment × week interactions (FDR-adjusted *p* = 0.024 for both). The model for PWY-5181 showed a singular fit, whereas no convergence or singular-fit problem was detected for PWY-7431. For PWY-5181, predicted abundance increased from week 1 to week 4 in both paired control cows, whereas it decreased in all three RPN cows. For PWY-7431, predicted abundance markedly increased in both paired control cows, whereas changes among RPN cows were smaller and varied in direction.

### 3.3. Exploratory Integrated Analysis of Uterine Arterial Hemodynamics, Microbiome Structure, and Predicted Metabolic Pathways

Figure 5d shows the results of an integrated analysis, which was performed in RPN-treated cows using changes (Δ) in uterine arterial resistance index (ΔRI), uterine microbiome structure (ΔAxis1), and predicted pathway abundance (ΔPWY-5181). All three RPN cows showed an increase in RI accompanied by a decrease in the predicted abundance of PWY-5181. Changes in PWY-5181 also showed a negative linear relationship with changes in PCoA Axis 1, linking changes in uterine microbiome structure with changes in predicted functional potential. When the corresponding ΔRI was incorporated graphically as a third dimension, the integrated pattern showed concurrent within-animal changes across uterine arterial hemodynamics, microbiome structure, and predicted functional potential.

## 4. Discussion

This study examined the effects of RPN supplementation on uterine hemodynamics and the endometrial environment during the postpartum period in Japanese Black cows using complementary acute and chronic experimental designs. In Experiment 1, high-dose RPN administration was associated with acute changes in uterine arterial diameter and vascular resistance, together with an overall increase in baseline-adjusted whole-blood NAD^+^ concentrations during the post-administration period. Experiment 2 evaluated the longer-term consequences of postpartum RPN supplementation and identified alterations in uterine blood flow indices, while exploratory microbiome analyses generated hypotheses regarding potential changes in the endometrial microbiota and its predicted functional potential. Exploratory integrated analysis further revealed associations between changes in uterine arterial resistance, microbial community structure, and predicted pathway abundances in RPN-supplemented cows. These findings suggest that postpartum RPN supplementation modulates uterine hemodynamics and these hemodynamic changes occur in association with selective alterations in the endometrial microbiome and its inferred metabolic potential.

In Experiment 1, the overall increase in baseline-adjusted whole-blood NAD^+^ concentrations following acute RPN administration indicates that the high-dose supplementation was accompanied by a measurable systemic metabolic response. Niacin serves as a precursor for NAD^+^ biosynthesis and may therefore influence systemic cellular redox and energy metabolism. However, because circulating niacin and intermediary metabolites were not measured in the present study, the metabolic pathway linking RPN administration to the observed increase in whole-blood NAD^+^ cannot be directly established. Although the present study was not designed to determine whether this NAD^+^ response directly contributed to the observed uterine hemodynamic changes, its concurrent occurrence provides additional evidence of a physiological response to acute RPN administration. The uterine hemodynamic response therefore provides a common physiological context for interpreting the acute and postpartum experiments. In the present study, RPN supplementation was associated with changes in uterine hemodynamics, particularly indices reflecting vascular resistance and blood velocity. Previous Doppler studies in cattle have demonstrated that uterine arterial RI and blood velocity vary with reproductive status, with higher RI being accompanied by lower blood velocity under conditions of reduced uterine arterial blood flow [17]. In the present study, the treatment-dependent changes in TAMV and RI therefore suggest modulation of uterine arterial hemodynamics rather than direct evidence of increased uterine perfusion. PVD scores also decreased over the postpartum period in both groups, broadly consistent with postpartum uterine recovery; however, the present data do not provide evidence that this change was specifically attributable to RPN supplementation. Treatment-associated changes in overall endometrial microbial diversity and community structure were limited, with more selective alterations observed in specific microbial taxa and predicted functional pathways. After parturition, the uterus of cows undergoes marked morphological, structural, and functional changes, eventually restoring its pre-pregnancy state, a process that is essential for the resumption of the estrous cycle and normal fertility in postpartum cows [27]. After parturition, the uterus is exposed to tissue damage and intense inflammatory responses [28], which are accompanied by a transient hypoxic state [29]. Under such conditions, both uterine tissues and the endometrial microbiota are expected to experience substantial metabolic and oxidative stress [29].

The endometrial microbiome did not show broad treatment-associated restructuring despite changes in uterine hemodynamics. This relative compositional stability suggests that postpartum nutritional modulation does not necessarily induce large-scale taxonomic reorganization of the uterine microbiota. Rather, the endometrial microbial community may be relatively resilient at the compositional level during early postpartum recovery, even under altered host physiological conditions. However, the observed variation in microbial community structure and predicted functional profiles suggests that predicted functional potential of the microbiome may vary without overt taxonomic replacement. Such predicted functional or structural variation may be associated with host-mediated changes in the local uterine environment. This observation is consistent with the concept of functional redundancy in mucosal microbiomes, whereby microbial communities can maintain a relatively stable taxonomic structure while undergoing substantial shifts in metabolic activity and functional output [30]. In mucosal tissues, including the reproductive tract, microbial function rather than species composition per se is increasingly recognized as a primary determinant of host–microbiome interactions and immune modulation [31]. Following parturition, the bovine uterus is characterized by tissue damage, strong inflammatory responses, and transient hypoxia associated with uterine involution and microbial clearance [29]. Such changes in tissue perfusion, oxygen tension, and inflammatory mediators are known to act as powerful ecological filters that shape microbial metabolism without necessarily causing large-scale changes in community composition. Experimental and clinical studies in mucosal systems have demonstrated that altered oxygen availability and inflammation may modulate or reprogram microbial energy metabolism and redox pathways, even within a relatively stable microbial community [32,33]. Thus, the compositional stability observed here could be compatible with functionally meaningful variation in predicted microbial functional potential, raising the hypothesis that such variation may be associated with RPN-related changes in uterine hemodynamics and the local physiological environment.

Although RPN supplementation did not produce broad treatment-associated changes in overall microbial diversity or community structure, the predicted functional analysis identified exploratory treatment-dependent temporal differences in two pathways, PWY-5181 and PWY-7431. PWY-5181 represents an aerobic toluene degradation pathway that enables bacteria to convert toluene into metabolites that can enter the tricarboxylic acid (TCA) cycle, thereby linking aromatic compound degradation to microbial energy metabolism [34,35]. PWY-7431 is involved in bacterial degradation of aromatic biogenic amines. Thus, although these pathway annotations represent distinct metabolic processes, both are related to the bacterial processing of aromatic compounds, suggesting that the predicted functional differences observed in this exploratory analysis may involve specific aspects of microbial metabolic potential rather than broad restructuring of the endometrial microbial community. Importantly, these functions were inferred from 16S rRNA gene profiles using PICRUSt2 and therefore represent predicted metabolic potential rather than directly measured pathway activity or metabolite concentrations.

The exploratory integrated analyses showed concurrent within-animal changes in uterine hemodynamics, microbial community structure, and predicted metabolic pathway abundance in the endometrial microbiome. These concurrent patterns raise the hypothesis that selective variation in predicted microbial functional potential may be associated with changes in host uterine physiology without extensive restructuring of the microbial community. In RPN-treated cows, the predicted abundance of PWY-5181 decreased consistently from week 1 to week 4, while uterine arterial RI increased in all three animals, and changes in PWY-5181 showed a negative relationship with changes in microbial community structure along PCoA Axis 1. When considered together, these observations represent concurrent within-animal patterns across uterine arterial hemodynamics, microbiome structure, and predicted microbial functional potential, which may generate hypotheses for subsequent studies. Because local blood flow influences the delivery of oxygen and nutrients and contributes to the physicochemical environment of tissues, modulation of uterine hemodynamics provides a plausible host physiological context in which changes in the endometrial microbial ecosystem may occur [4]. Although these integrated observations should be regarded as exploratory and hypothesis-generating, they suggest that RPN supplementation may be associated with uterine hemodynamics and selective changes in the predicted functional potential of the endometrial microbiome and that these changes may be interrelated during postpartum uterine recovery.

Several limitations of this study should be acknowledged. First, the small number of animals limits statistical power, particularly for microbiome and multivariate analyses, and increases the influence of inter-individual variability and the risk of model instability or overfitting. This limitation is particularly relevant to Experiment 2 (*n* = 3 per group), and the loss of one control microbiome sample at week 1 further reduced the available sample size. Second, Doppler acquisition and offline image analysis were not fully blinded to treatment allocation in both Experiments, and potential measurement bias cannot be completely excluded, although standardized acquisition and analysis procedures were applied throughout the study. In addition, Doppler angle correction was not applied for TAMV measurements; therefore, the absolute TAMV values should be interpreted cautiously despite the standardized acquisition procedures. Third, functional profiles of the endometrial microbiota were inferred using PICRUSt2 and therefore represent predicted metabolic potential rather than directly measured microbial activity. Likewise, differential abundance analysis based on 16S rRNA gene sequencing should be interpreted cautiously, particularly for low-abundance taxa. In addition, endometrial microbiome samples can contain low microbial biomass, which may increase analytical uncertainty, particularly because an extraction blank was not included in the original experimental design. A PCR negative control was included during library preparation. No detectable amplification was observed, and therefore the negative control was not subjected to sequencing. Nevertheless, contamination-related signals cannot be confidently excluded for low-prevalence taxa, particularly those showing very large estimated fold changes in the exploratory DESeq2 analysis. Fourth, the integrated analysis was exploratory and identified associations rather than causal or mechanistic relationships among uterine hemodynamics, microbial community structure, and predicted metabolic pathways. Because this analysis was restricted to the three RPN-treated cows, the observed relationships should be interpreted as within-group patterns that require confirmation in larger cohorts. Finally, this study was conducted in Japanese Black cattle during the early postpartum period, and the generalizability of these findings to later postpartum stages or other breeds remains unknown.

## 5. Conclusions

RPN supplementation modulated uterine hemodynamics during the postpartum period and was accompanied by selective changes in the endometrial microbiome and its predicted functional potential. The concurrent changes observed across uterine arterial hemodynamics, microbial community structure, and predicted microbial functions suggest that uterine hemodynamics may represent a physiological interface associated with nutritional modulation of the postpartum endometrial environment. These findings provide a basis for further investigating the interplay between nutritional status, uterine circulation, and the endometrial microbial ecosystem, including the hypothesis that these interactions may be relevant to subsequent reproductive physiology in beef cattle.

## Data Availability

The raw sequencing data have been deposited in the NCBI Sequence Read Archive (SRA) under BioProject accession number PRJNA1431663.

## Supplementary Materials

The following supporting information can be downloaded at https://www.mdpi.com/article/10.3390/ani16203235/s1: Supplementary Method: *16S rRNA* gene sequencing, Table S1: Prevalence and individual-sample raw counts of taxa showing FDR-adjusted significance in DESeq2 analyses.
