# Supplementary material for: Rumen-Protected Niacin Supplementation Modulates Uterine Arterial Hemodynamics and Endometrial Microbiome Functional Potential in Postpartum Japanese Black Cattle
Source: Animals (Basel). 2026 Oct 14;16(20):3235. doi: 10.3390/ani16203235 (PMC13700562; doi:10.3390/ani16203235)
Supplement: Supplementary file 1 [file animals-16-03235-s001.zip › animals-4571743-supplementary/animals-4571743-supplementary.pdf]

Supplementary Methods

16S rRNA gene sequencing

Collected endometrial smear samples were stored at −30 °C and sent to Seibutsu Giken Inc. (Kanagawa, Japan) for DNA extraction and NGS sequencing. The samples were mixed with Lysis Solution F (Nippon Gene Co., Ltd., Tokyo, Japan) and disrupted using a Shake Master Neo (Biomedical Science Co., Ltd., Tokyo, Japan) at 1,500 rpm for 2 min. The disrupted samples were incubated at 65 °C for 10 min and then centrifuged at 12,000 × g for 2 min. DNA was purified from the collected supernatant using the MPure-12 system and MPure Bacterial DNA Extraction Kit (MP Biomedicals).

The library was prepared from the purified samples using a 2-step tailed PCR method. The V3–V4 region of the bacterial 16S rRNA gene was amplified using primers based on Klindworth et al. (2013). The first-step primers were 1st-341f_MIX (5′-ACACTCTTTCCCTACACGACGCTCTTCCGATCT-NNNNN-CCTACGGGNGGCWGCAG-3′) and 1st-805r_MIX (5′-GTGACTGGAGTTCAGACGTGTGCTCTTCCGATCT-NNNNN-GACTACHVGGGTATCTAATCC-3′), and the second-step primers were 2ndF(5’-AATGATACGGCGACCACCGAGATCTACAC-Index2-ACACTCTTTCCCTACACGACGC-3’) and 2ndR (5’- CAAGCAGAAGACGGCATACGAGAT-Index1-GTGACTGGAGTTCAGACGTGTG-3’). The first PCR was performed in a total volume of 10 μL containing 1.0 μL of 10× Ex Buffer, 0.8 μL of dNTPs (each 2.5 mM), 0.5 μL of each primer (10 μM), 2.0 μL of template DNA, 0.1 μL of ExTaq HS (5 U/μL; Takara Bio, Shiga, Japan), and 5.1 μL of sterile ultrapure water. The second PCR used the same reaction mixture, except that 2.0 μL of the first PCR product was used as the template. The first PCR conditions were initial denaturation at 94 °C for 2 min, followed by 30 cycles of 94 °C for 30 s, 55 °C for 30 s, and 72 °C for 30 s, with a final extension at 72 °C for 5 min. The second PCR conditions were initial denaturation at 94 °C for 2 min, followed by 10 cycles of 94 °C for 30 s, 60 °C for 30 s, and 72 °C for 30 s, with a final extension at 72 °C for 5 min. A PCR negative control containing sterile ultrapure water instead of template DNA was included.

The concentration of the prepared libraries was measured using a microplate fluorescence reader (Synergy H1, BioTek, Winooski, VT, USA) and QuantiFluor dsDNA System (Promega, Madison, WI, USA) following the manufacturer’s instructions. The quality of the prepared libraries was examined using Fragment Analyzer and dsDNA 915 Reagent Kit (Agilent Technologies). Sequencing was performed using the MiSeq system with MiSeq Reagent Kit v3 (Illumina, San Diego, CA, USA) to generate 2 × 300 bp paired-end reads.

Reads exactly matching the primer sequences were extracted using fastx_barcode_splitter in FASTX-Toolkit v0.0.14, and primer sequences were removed using fastx_trimmer. Reads with quality scores <20 and reads shortened to ≤130 bp, together with their paired reads, were discarded using Sickle v1.33. Paired-end reads were subsequently merged using FLASH v1.2.11. Sequencing generated 56,227–77,412 paired-end read pairs per sample. After quality filtering and paired-read merging, 7,716–31,218 merged sequences per sample were obtained (median, 13,903). Preprocessed sequences were then analyzed using Quantitative Insights Into Microbial Ecology 2 (QIIME 2; v2021.4). Chimeric and noisy sequences were removed using the DADA2 plugin, and representative sequences and an amplicon sequence variant (ASV) table were generated. Taxonomic classification was performed using the feature-classifier plugin by comparing the representative sequences against the Greengenes database (v13_8) 97% OTU reference sequences. Sequence alignment and phylogenetic tree construction were performed using the alignment and phylogeny plugins, respectively.

For differential abundance analysis, the ASV table was taxonomically aggregated at the genus level, and the resulting genus-level abundance table was used for DESeq2 analysis. For prediction of microbial functional potential, the ASV-level feature table and corresponding representative sequences were directly used as input for the PICRUSt2 full pipeline (q2-picrust2, QIIME 2 v2024.5). During PICRUSt2 processing, 53 input ASVs were excluded because less than 80% of their sequences aligned to the reference sequences (min_align = 0.8), and one ASV was subsequently excluded because its nearest-sequenced-taxon index (NSTI) exceeded the default cutoff of 2.0. The resulting predicted pathway abundance table was used for downstream pathway analysis.
